# Do Control Beliefs Moderate the Relationship Between Function and Physical Activity?

**DOI:** 10.1093/geroni/igaa057.1290

**Published:** 2020-12-16

**Authors:** Elizabeth Teas, Elliot Friedman

**Affiliations:** Purdue University, West Lafayette, Indiana, United States

## Abstract

Most older adults get far less than recommended levels of physical activity (PA), and interventions to improve PA have limited effectiveness. Barriers to PA include reduced physical function (PF) and diminished feelings of control, but their interactive influences on PA in older adults are unclear. Using two methodologies, the current study determined whether control beliefs modify the relationship between PF and PA. Data were from the second wave of the Midlife in the United States (MIDUS) study; the sample was constrained to participants with PA greater than 0 (n= 955, mean age= 54.27). PF (grip strength, gait speed, and chair stands) was measured during a clinic visit. Participants were asked about routine PA, from which a Metabolic Equivalent of Task score was calculated, and the extent to which they believed they have control over their life (0-7 scale). In linear regression models, including interactions between control beliefs and PF variables, gait speed and control were associated with PA; none of the interaction terms were significant. The second model used a person-centered approach to explore the potential of non-linear relationships and differences in groups of people by creating typologies. The group with low control and slow gait speed had significantly lower PA than the other three groups. Results suggest nuanced associations among PA, PF, and control beliefs where feelings of control may compensate for slow gait speed in particular. They also support the use of person-centered approaches to identify non-linear associations between modifiable protective factors and key outcomes in aging research.

